# Emergence of genetically linked vaccine-originated poliovirus type 2 in the absence of oral polio vaccine, Jerusalem, April to July 2022

**DOI:** 10.2807/1560-7917.ES.2022.27.37.2200694

**Published:** 2022-09-15

**Authors:** Neta S Zuckerman, Itay Bar-Or, Danit Sofer, Efrat Bucris, Hagar Morad, Lester M Shulman, Nofar Levi, Leah Weiss, Irina Aguvaev, Zvi Cohen, Klil Kestin, Rinat Vasserman, Michal Elul, Ilana S Fratty, Miranda Geva, Marina Wax, Oran Erster, Ruth Yishai, Lior Hecht-Sagie, Sharon Alroy-Preis, Ella Mendelson, Merav Weil

**Affiliations:** 1 Central Virology Laboratory, Public Health Services, Ministry of Health, Chaim Sheba Medical Center, Ramat Gan, Israel; 2 School of Public Health, Sackler Faculty of Medicine, Tel-Aviv University, Tel-Aviv, Israel; 3 The Israel Center for Disease Control, Israel Ministry of Health, Ramat-Gan, Israel; 4 Department of Laboratories, Public Health Services, Ministry of Health, Jerusalem, Israel; 5 Public Health Services, Ministry of Health, Jerusalem, Israel

**Keywords:** Vaccine Derived Polio Virus (VDPV), Polio type 2 (PV2), Environmental surveillance, Whole Genome Sequencing (WGS)

## Abstract

We report an emergence and increase in poliovirus type 2 detection via routine wastewater surveillance in three non-overlapping regions in the Jerusalem region, Israel, between April and July 2022. Sequencing showed genetic linkage among isolates and accumulation of mutations over time, with two isolates defined as vaccine-derived polioviruses (VDPV). This demonstrates the emergence and potential circulation of type 2 VDPV in a high-income country with high vaccine coverage and underscores the importance of routine wastewater surveillance during the polio eradication.

Routine acute flaccid paralysis (AFP) surveillance for poliovirus (PV) in Israel has been enhanced by supplementary wastewater environmental surveillance (ES) since 1989 [1]. Sabin-like PV types 1 and 3 are detected in most ES samples because bivalent oral poliovirus vaccine (OPV) is used in Israel routine immunisations. Since the discovery of circulating vaccine-derived poliovirus (cVDPV) type 3 from the end of 2021 throughout 2022 [2], sampling frequency and sites were increased in the Jerusalem region and elsewhere. Here, we describe frequently increasing detections of PV2 in the Jerusalem region in the absence of OPV type 2 immunisations and examine the genetic linkage across these detections.

**Environmental surveillance monitoring sites**

The ES monitors 15 sites across the country once a month, with a catchment of ca 50% of the population in Israel [1]. The Jerusalem region includes four non-overlapping ES sampling sites, three of them located at the inlets of wastewater treatment plants and one in the Kidron River that collects wastewater from central and south-east Jerusalem (Figure 1A). 

**Figure 1:**
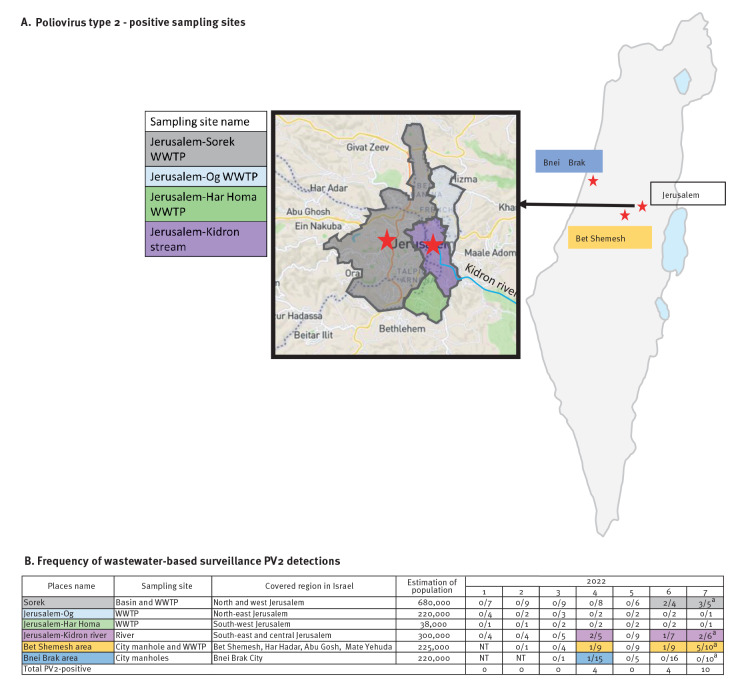
Environmental detections of poliovirus type 2, Israel, 2022

NT: not tested; PV2: poliovirus type 2; WWTP: wastewater treatment plant.

^a^ Samples still under processing.

Panel A: Red stars indicate the geographical locations of PV2-positive detections in Israel. The Jerusalem region is magnified to show the specific regions being sampled in environmental surveillance and where PV2 was detected.

Panel B: Sampling sites with PV2-positive detections only are indicated, including catchment area, description of region coverage, frequency of detection in each site (nominator is the number of PV2-positive samples; denominator is the total number of samples examined) and the total number of PV2-positive samples identified overall in each month, January–July 2022.

## Detection of poliovirus type 2 vaccine and vaccine-derived virus in wastewater collected from the Jerusalem region

**Figure 2:**
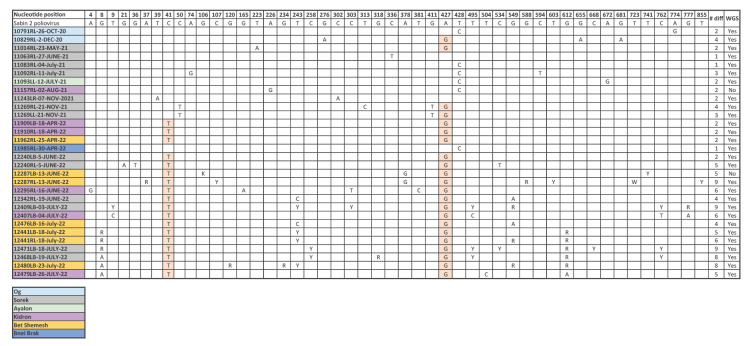
Nucleotide changes in the VP1 gene of poliovirus type 2 wastewater isolates, Israel, 2020–2021 (n = 11)

VDPV: vaccine-derived poliovirus; WGS: whole genome sequencing; # diff: number of mutations.

In April 2022, PV2 was detected in three sampling sites – two in the Jerusalem region (Kidron, Bet Shemesh) and one in Bnei Brak in central Israel (Figure 1A). In June, PV2 was detected in three sampling sites in the Jerusalem region (Kidron, Bet Shemesh and Sorek, Figure 1A). In July, we observed a substantial increase in the number of PV2 detections in the Jerusalem region (n = 10, Figure 1B), although the final number of PV2-positive samples in July may have been higher as some isolations were still under processing at the time of publication of this article. The PV2 isolates detected during 2022 by RT-PCR were partially sequenced using the Sanger method (VP1 gene, 903 nt) [3] and compared with the Sabin 2 vaccine strain reference sequence (AY.184220) along with VP1 sequences from all PV2 previously detected in ES during 2020 and 2021 in Israel (n = 11, Figure 2). We observed an accumulation in nucleotide changes in sequences from June and July 2022, classifying two isolates as VDPV type 2 (defined as ≥ 6 nt changes from Sabin 2 [4]). Five additional isolates with ≥ 6 nt changes including mixed bases were not classified as VDPV type 2. Most importantly, PV2 sequences collected in 2022 shared a 2 nt genetic signature in the VP1 gene: C41T, which was not observed in any of the sequences from 2020 and 2021, nor in the Bnei Brak sequence, and A427G, which is a reversion of one of two attenuation sites in the Sabin 2 strain and was also identified in two of the sequences from 2020 and 2021.

The VP1 gene (903 nt) was sequenced using Sanger and compared with the Sabin 2 strain to detect mutations. Changes of ≥ 6 nt indicate a VDPV2. Nucleotide positions with mixed bases were not counted towards the ≥ 6 nt changes needed to designate an isolate as a VDPV2. L20B (LB, mouse L cells transfected with a gene for the human cellular receptor for poliovirus) and RD (derived from a human rhabdomyosarcoma) cell cultures were used for poliovirus isolation according to the World Health Organization protocol [1]. The LB (L20B), RL (RD passage to L20B), LR (L20B passage to RD) and LL (L20B passage to L20B) added to the sample names indicate the type of the tissue culture and passage used to isolate PV2.

Sample names are coloured by the region in which they were detected as indicated in the legend. Mutations highlighted in orange are mutations which are common to all 2022 isolates. Mixed bases are indicated as: R = A/G; Y = C/T; K = G/T; W = A/T.

## Identification of genetic signatures linking the 2022 PV2 isolates by whole genome sequencing

**Figure 3:**
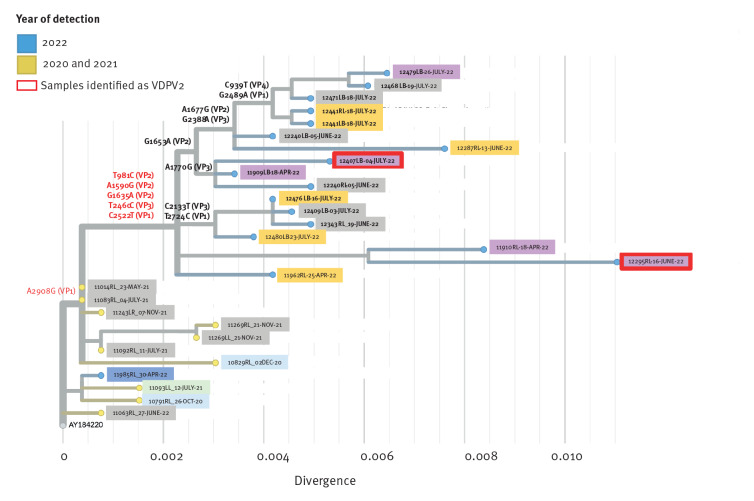
Phylogenetic analysis of the P1 region of polioviruses wastewater isolates, Israel, 2020–2022 (n = 28)

To further explore the genetic linkage between the isolates, we performed whole genome sequencing (WGS) for isolates from 2020 to 2022 (Figure 2). Libraries were prepared with SMARTer Stranded RNA-Seq (Takara Bio, United States (US)) and sequenced on the NovaSeq 6000 system (Illumina, US). Fastq files were aligned against the P1 region (this includes the VP1, 2, 3 and 4 genes) in all poliovirus Sabin strains and only sequence reads matching Sabin 2 were retained. The rest of the genome (P2/3 region) was assembled de novo (using SPADES [5]) because this genomic region has high similarities across enteroviruses and a tendency to undergo recombination with other C-species enteroviruses [6]. We assembled the P1 and P2/3 segments via alignment to the Sabin 2 whole genome (GenBank accession numbers are still pending at the time of publication and will be provided in an Addendum once available). Phylogenetic analysis via Nextstrain augur pipeline [7] and mutational analyses of the P1 region revealed additional mutations shared across all 2022 sequences that were not detected in the sequences from Bnei Brak or those from 2020 and 2021: T981C (VP2), A1590G (VP2), G1635A (VP2), T2460C (VP3) (Figure 3). 

VDPV: vaccine-derived poliovirus.

High resolution phylogenetic tree of the P1 region (includes the VP1, 2, 3 and 4 genes) representing genomic differences across the samples. Shared mutation locations relative to the whole genome are indicated in the tree for splits representing three or more samples.

The expanded signature mutations shared across all the 2022 strain are coloured in red.

**Figure 4:**
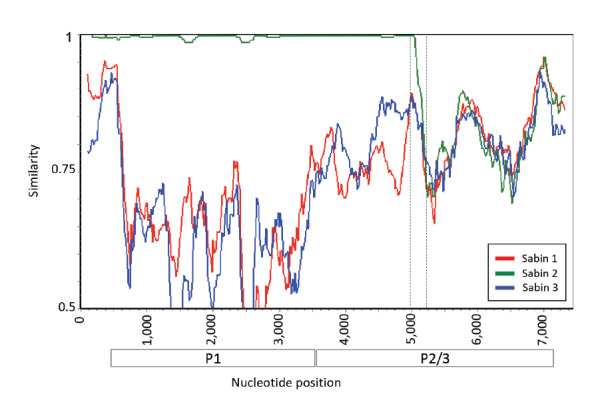
Recombination event in poliovirus 2 sequences from wastewater isolates, Israel, 2022 (n =28)

Interestingly, the assembled P2/3 genomic region was not well aligned with any of the Sabin strain genomes, suggesting a recombination in this region with a non-poliovirus enterovirus. This unique recombination, occurring in the 3A gene (nucleotide position ca 5,100 in the genome), was present in all 2022 sequences but not in the 2022 Bnei Brak sequence or any of the sequences from 2020 and 2021, further strengthening the genetic linkage across the 2022 sequences (Figure 4).

Simplot [8] of the Sabin 1, 2 and 3 strains compared with 2022 poliovirus 2 isolates demonstrates highest similarity to Sabin 2 (AY.184220) in the P1 region. Similarity to Sabin 2 declines in the P2/3 region at a position around 5,100 (dashed vertical lines), with low similarity of the 2022 PV2 isolates to all three Sabin strains, suggesting recombination with a non-polio enterovirus.

## Discussion

Vaccine-derived polioviruses originate from genetically mutated OPV (types 1, 2, or 3 Sabin strains) that accumulate mutations; reversions in vaccine attenuation sites may lead to neurovirulence of such strains [4]. This process usually occurs during extensive person-to-person transmission in populations with low immunity (circulating cVDPV) or following extended replication in the intestine and/or oropharyngeal mucosa of an immunodeficient vaccinated individual (immunodeficient iVDPV) [4].

As cVDPV type 2 caused most VDPV outbreaks, OPV type 2 was removed globally in April 2016 from the routine trivalent OPV which now contains only types 1 and 3 (bivalent OPV) [9]. Since then, monovalent OPV2 has been given only as a response to cVDPV2 outbreaks [10]. Nevertheless, an increase in the number of cVDPV type 2 outbreaks has been reported worldwide in recent years [11]. During 2022, a new challenge in the World Health Organization (WHO) poliovirus eradication programme includes controlling PV2 events not only in low-income countries [12] but also in countries with high vaccination coverage such as the US (New York), the United Kingdom and Israel [13].

Here we report for the first time an increase in the frequency of PV2 isolates detected in the Jerusalem region in Israel via wastewater surveillance. Sequencing of PV2 showed genetic linkage across the isolates and accumulation of mutations over time, with two isolates defined as VDPV. These PV2 isolates were detected in three different non-overlapping wastewater catchment regions in the Jerusalem region over a period of 100 days, which makes it unlikely that these isolates originated from one individual. We therefore assume that this VP2 strain might be circulating in this region, although not currently defined as such by the WHO. Genetically linked PV2 isolates were also identified in New York and London [14]. As monovalent OPV2 is not given in routine immunisations in these countries, these isolates may have originated from countries holding OPV2 campaigns due to recent VDPV2 outbreaks. Interestingly, in 2020 and 2021, importations of PV2 strains to Israel did not result in prolonged, evolving circulation, and those strains disappeared. The establishment of this PV2 strain from 2022 in Israel started during a cVDPV3 outbreak in the same region. The subsequent emergence of two such rare events is unexpected, especially in a high-income setting with high vaccination coverage. Israel responded to the first event (cVDPV3) by administrating an inactivated poliovirus (IPV) including inactivated PV2 and a bivalent OPV that does not include Sabin 2. Thus, the vaccination programme cannot prevent transmission of PV2 but can prevent the disease in vaccinated individuals. As per July 2022, transmission of this PV2 strain in Israel has remained local and has not been detected outside the Jerusalem region, nor have any AFP cases been caused by PV2. Despite the high vaccination coverage in Jerusalem, there are still sub-populations with lower coverage in this region. As a response to the recent PV2 findings, the Israeli Ministry of Health initiates specialised programmes to vaccinate every child with IPV to prevent the disease, specifically targeting susceptible under-vaccinated communities in Jerusalem that were identified during the earlier emergence of cVDPV3. The early detection and identification of the genetic linkage across the isolates of this strain were based on WGS and extensive wastewater surveillance with advanced technologies. Indeed, with only two mutations in the routinely Sanger-sequenced VP1 gene linking the isolates (one of which was also observed in PV2 sequences from 2020 and 2021), WGS strengthened the genetic linkage by identifying additional mutations and a unique recombination shared across the 2022 isolates. Taking this case as an example, characterisation methods and working protocols may require updates in order to improve resolution of identification and response time to future outbreaks. 

## Conclusion 

This event emphasises the importance of routine environmental/AFP surveillance as a means for outbreak control and rapid immunisation campaigns, not only in low-income countries, but also in high-income countries with high vaccination coverage.
